# Relations between recurrence risk perceptions and fear of cancer recurrence in breast cancer survivors

**DOI:** 10.1007/s10549-022-06684-3

**Published:** 2022-07-30

**Authors:** J. W. Ankersmid, F. K. Lansink Rotgerink, L. J. A. Strobbe, C.F. van Uden-Kraan, S. Siesling, C. H. C. Drossaert

**Affiliations:** 1 Department of Health Technology and Services Research, Technical Medical Centre, University of Twente, Enschede, The Netherlands; 2 Santeon, Utrecht, The Netherlands; 3 Department of Surgery, Canisius Wilhelmina Hospital, Nijmegen, The Netherlands; 4 Department of Research and Development, Netherlands Comprehensive Cancer Organisation (IKNL), Utrecht, The Netherlands; 5 Department of Psychology, Health & Technology, University of Twente, Enschede, The Netherlands

**Keywords:** Breast cancer, Survivorship, Risk information, Recurrences, Fear of cancer recurrence

## Abstract

**Purpose:**

This study aimed to: (1) determine the accuracy of Dutch breast cancer survivors’ estimations of the locoregional recurrence risk (LRR); (2) examine which variables influence (the accuracy of) risk estimations, and risk appraisals; and (3) investigate the influence of the objective LRR risk (estimated using the INFLUENCE-nomogram), risk estimations and risk appraisals on fear of cancer recurrence (FCR). Findings of this study will inform clinicians on risk communication and can improve communication about FCR.

**Methods:**

In a cross-sectional survey among 258 breast cancer survivors, women’s recurrence risk estimations (in odds) and risk appraisals (in high/low), FCR, demographics and illness perceptions, about one year after surgery were measured and compared to the objective risk for LRRs estimated using the INFLUENCE-nomogram.

**Results:**

Half of the women (54%) accurately estimated their LRR risk, 34% underestimated and 13% overestimated their risk. Risk estimations and risk appraisals were only moderately positively correlated (*r* = 0.58). Higher risk appraisals were associated with radiotherapy (*r* = 0.18) and having weaker cure beliefs (*r* = − 0.19). Younger age was associated with overestimation of risk (*r* = − 0.23). Recurrence risk estimations and risk appraisals were associated with more FCR (*r* = 0.29, *r* = 0.39). In regression, only risk appraisal contributed significantly to FCR.

**Conclusion:**

Although women were fairly accurate in recurrence risk estimations, it remains difficult to predict over- or underestimation. Recurrence risk estimations and risk appraisal are two different concepts which are both associated with FCR and should therefore be addressed in patient-provider communication.

## Introduction

The incidence of and survival rates after breast cancer have been rising in the Netherlands, which led to an increase in the number of survivors receiving follow-up care [1]. Follow-up care consists of *aftercare* and *post-treatment surveillance*. *Aftercare* focusses on information provision, guidance, identification of and dealing with complaints, symptoms and physical and psychosocial effects of the condition and treatment [2]. *Surveillance* is primarily aimed at early detection of locoregional recurrences (LRRs) and second primary tumours (SPs) [2].

Unlike the highly personalised treatment, post-treatment surveillance is currently one-size-fits-all. However, research shows that risks for LRRs and SPs differ for every patient (depending on patient-, tumour- and treatment characteristics), change over time and are generally low [3]. The INFLUENCE-nomogram is a prediction model which enables the estimation of individual risks for LRRs and SPs within the first 5 years after surgery based on patient-, tumour and treatment characteristics [4]. This model can be used to identify patients who might benefit from less or more intensive surveillance and to inform patients about their personal risks within the process of shared decision-making (SDM) about personalised surveillance [5].

For SDM about personalised surveillance it is essential that patients understand their recurrence risk. Liu et al. found that only few breast cancer survivors estimated their recurrence risk accurately [6]. Misunderstanding of recurrence risks can have broad consequences. Overestimation of recurrence risk can lead to more frequent worry, higher anxiety levels, or a lower quality of life [7–10]. Underestimation may, in its turn, negatively affect adherence to surveillance recommendations or cause health care avoidance [11].

Besides the absolute risk perception, which is usually expressed in numbers (e.g., 1 in every 50 women), other risk perception types exist, such as risk appraisal (i.e., whether women appraise their risk as high, low or average). These two types are probably related, but as far as we know no studies have examined their relationship in breast cancer survivors.

Existing studies report several factors that are associated with risk perceptions and over- and underestimation of recurrence risks in breast cancer survivors such as: age; illness perceptions; patient-provider communication; receiving radiotherapy; social support; spirituality, religion and faith; uncertainty; and family and personal history [9, 11–16]. However, most of these studies focus on other types of risks than the risk for LRR (e.g., distant metastasis or contralateral breast cancer risks). Furthermore, risk perceptions are often measured immediately after diagnosis or during active treatment instead of during the follow-up trajectory. Most studies also determine the accuracy of risk based on general risk and not based on personalised risk estimations.

Studies on the relationship between risk perceptions and FCR are scarce. The studies that do exist state that overestimation of recurrence risk is associated with increased FCR [6, 9, 15–17]. With increased understanding of how risk perceptions regarding LRR risks influence levels of FCR among survivors of invasive early-stage breast cancer, information provision, and supportive care after cancer can be improved [18].

**Fig. 1:**
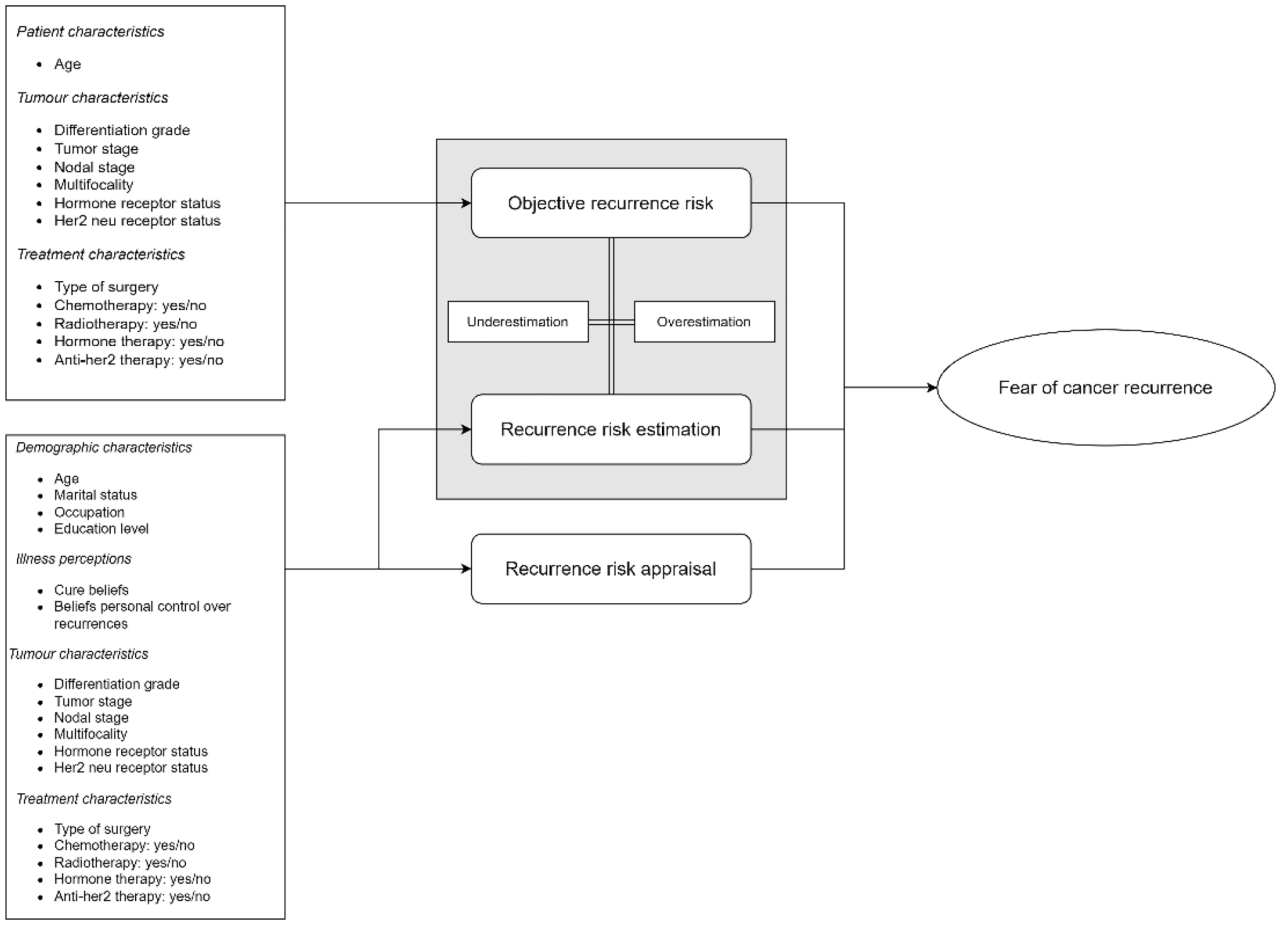
Conceptual framework

Based on the variables arising from the beforementioned literature, the authors developed a conceptual framework in order to address this study’s research questions (Fig. 1).

This study aimed to: (1) determine the accuracy of Dutch breast cancer survivors’ estimations of the LRR risk; (2) examine which variables influence (the accuracy of) risk estimations, and risk appraisals; and (3) investigate the influence of the objective LRR risk (estimated using the INFLUENCE-nomogram), risk estimations, and risk appraisals on FCR.

## Methods

### Study setting

For this study, a subset of data of pre-implementation phase of the SHOUT-BC study (SHared decision-making supported by OUTcome information regarding Breast Cancer follow-up, Netherlands Trial Registry nr. NL8374) was used. The SHOUT-BC study started in November 2019 and investigates the implementation and efficacy of SDM supported by outcome information about personalised surveillance. The SHOUT-BC study takes place in the seven Santeon hospitals, a group of Dutch teaching hospitals that collaborate to improve care, and one other Dutch teaching hospital.

### Data collection

#### Participants and procedures

Patients attending one of the eight hospitals for their first surveillance consultation about one year after surgery were invited to participate in the study by their health care professional. Patients were eligible if they were 18 years or older; received curative treatment for invasive breast cancer; were able to give informed consent; and understood the Dutch language. Patients were not eligible if they were male; had been diagnosed with non-invasive breast cancer (e.g., DCIS); if this was their second diagnosis for breast cancer; if they received palliative treatment; neo-adjuvant systemic therapy; or if they had dementia. This study was conducted in accordance with local laws and regulations. The Medical research Ethics Committees United in Nieuwegein, the Netherlands, has confirmed that the Medical Research Involving Human Subjects Act (WMO) does not apply to this study (reference number W19.154). After providing informed consent, participants received a questionnaire (either online or on paper) around one year after their surgery. This moment was chosen because the questionnaire was part of a trial evaluating the implementation and efficacy of SDM supported by outcome information about personalised surveillance. The decision regarding personalised surveillance takes place around the first surveillance consultation about one year after surgery because for most patients active treatment has ended by that time.

#### Measures

Demographic characteristics, illness perceptions, recurrence risk estimations, recurrence risk appraisals, and fear of recurrence were measured using a questionnaire that was send to participants after the first surveillance consultation approximately one year after surgery. Demographic variables such as age, marital status, occupation, and education level were measured using general questions at the start of the questionnaire. Illness perceptions were measured using two domains of the Revised Illness Perceptions Questionnaire for breast cancer survivors (IPQ-BCS) [19]. The two domains consist of eight questions, four about cure beliefs (i.e., whether women believe that their breast cancer is cured) and four about personal control (i.e., the extent to which women believe to have control over cancer recurrence). The IPQ-BCS domains were translated from English to Dutch using a forwards-backwards translation procedure (following the COSMIN criteria [20]). All questions could be answered using a five-point Likert scale ranging from ‘strongly disagree’ to ‘strongly agree’. Scores were calculated by adding up the scores of the answers for each domain, after reversing the score on three items. Total scores per domain ranged from 0 to 16 with higher scores indicating stronger cure beliefs and higher personal control. Respondents current recurrence risk estimations were measured using the question *“How high do you estimate your risk of breast cancer recurrence in the same or the other breast?”*. Answering options were: 1 in 1000, 1 in 100, 1 in 50, 1 in 25, 1 in 10, and 1 in 5. Recurrence risk appraisals were measured using the question *“How do you rate your risk of breast cancer recurrence in the same or the other breast?”*. Participants were asked to appraise their risk of breast cancer recurrence on a five-point Likert scale ranging from ‘very low’ to ‘very high’. FCR was measured with the six-item Cancer Worry Scale (CWS) which is an validated instrument to detect FCR in (breast cancer) survivors [21]. The CWS scale was translated from English to Dutch using the same procedure as for the IPQ-BCS domains. All items were scored on a four-point Likert scale ranging from ‘never’ to ‘almost always’. Total scores ranged from 6 to 24 with higher scores indicating higher FCR. Scores of 12 or higher indicate that respondents experience high levels of FCR [21].

The objective risk for LRRs was estimated based on patient-, tumour- and treatment characteristics (derived from the Electronic Health Record) using the INFLUENCE-nomogram (version 2.0, https://www.evidencio.com/models/show/2238) [4]. The estimated objective risk for recurrences in combination with women’s risk estimations were used to determine accuracy of estimations.

### Data analysis

**Fig. 2:**
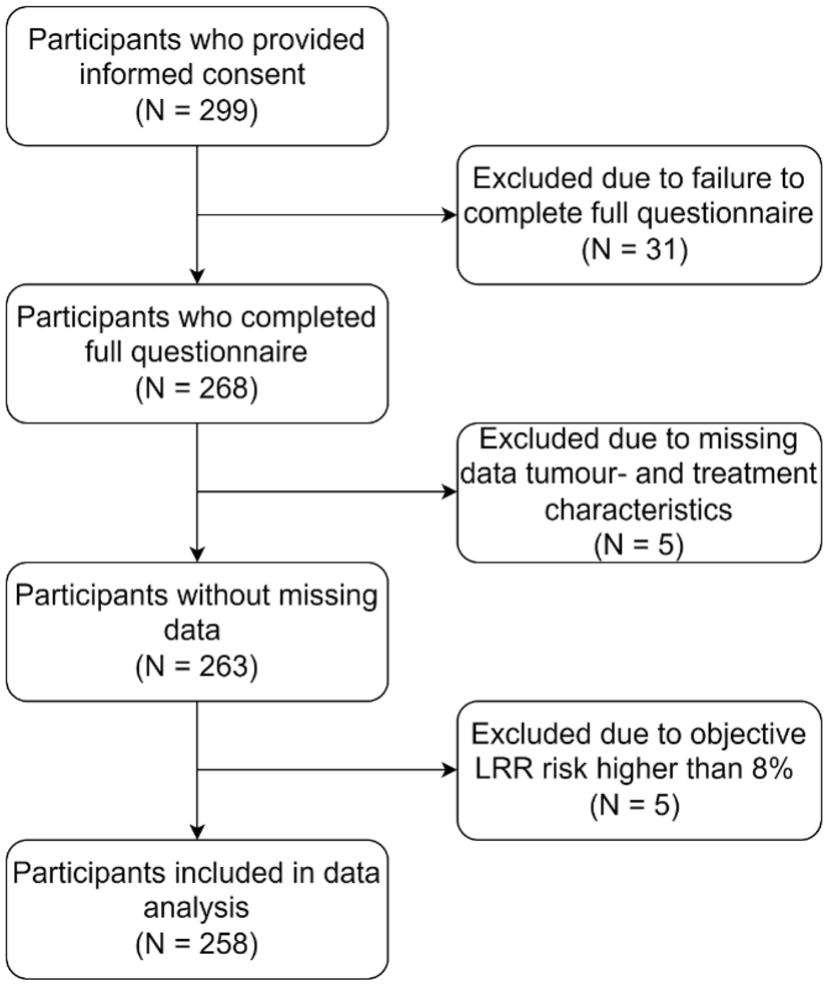
Participant flowchart

The participant flowchart is displayed in Fig. 2. Only patients who completed the questionnaire and for whom the data on the tumour- and treatment characteristics were complete were included in the data analysis. Participants with a LRR risk of eight percent or higher were excluded from the analysis, because their risk was more than four times the standard deviation away from the average risk, which made accurate estimation of their recurrence risk harder in comparison to other participants. Descriptive statistics were used to characterise the sample. We performed Spearman’s rank-order correlation analyses to assess the relationship between recurrence risk estimations and recurrence risk appraisals; to evaluate the relationship between several variables and recurrence risk estimation, recurrence risk appraisals, and accuracy of risk estimations; and to measure the association of LRR risks and risk estimations and appraisals with FCR. The significance level for these analyses was set at 0.01 due to the high amount of comparisons performed. A multiple regression analysis with a confidence interval of 0.98 was performed to predict FCR from the objective recurrence risk (estimated using the INFLUENCE-nomogram), risk estimations, and risk appraisals. All assumptions for this type of analysis were checked beforehand. All statistical analyses were performed in R version 4.1.0 (R Foundation for Statistical Computing, Vienna, Austria) and IBM SPSS Statistics (version 26).

## Results

### Sample description

In total, 258 participants were included in the analysis (Table 1). The mean age of participants was 59.4 years. Most participants were married (75.6%) and little under half of participants were employed (44.6%). Education levels of the participants were well-divided over higher, middle, and lower education. Most women had relatively small tumours and tumours with a differentiation grade of I or II. Most of these tumours were hormone receptor positive. Most participants had a lumpectomy as type of surgery (81.8%) and received radiotherapy (81.8%) and/or hormonal therapy (57.4%) as adjuvant therapy. Objective LRR risks of patients ranged from 1.3% to 7.5% with an average of 3%.

**Table 1:** Baseline characteristics (*n* = 258)

Demographics*	
Age, mean (*SD*), years	59 (10.4)
Married/partnership, *n* (%)	195 (75.6%)
Currently employed, *n* (%)	115 (44.6%)
Education level	
High, *n* (%)	104 (40.3%)
Middle, *n* (%)	63 (24.4%)
Low, *n* (%)	91 (35.3%)
Illness perceptions*	
Believe breast cancer is cured, *n* (%)	108 (41.9%)
Believe personal actions affect recurrence risk, *n* (%)	40 (15.6%)
Tumour characteristics**	
Differentiation grade (Bloom–Richardson), *n* (%)	
Grade I	86 (33.3%)
Grade II	130 (0.4%)
Grade III	42 (16.3%)
Tumour stage (pT stadium, pathological), *n* (%)	
T1	173 (7.1%)
T2	77 (29.8%)
T3	8 (3.1%)
Nodal stage (pN stadium, pathological), *n* (%)	
N0	192 (4.4%)
N1–3	61 (23.6%)
N > 3	5 (1.9%)
Multifocality, *n* (%)	29 (11.2%)
Hormone receptor (ER/PR) positive, *n* (%)	243 (94.2%)
Her2neu receptor positive, *n* (%)	11 (4%)
Treatment characteristics**	
Type of surgery, *n* (%)	
Lumpectomy	211 (1.8%)
Mastectomy	47 (18.2%)
Chemotherapy, *n* (%)	50 (19.4%)
Radiotherapy, *n* (%)	211 (81.8%)
Hormonal therapy, *n* (%)	148 (57.4%)
Trastuzumab, *n* (%)	11 (4%)
Locoregional recurrence risk estimate***	
Mean (SD), min–max	3% (1.3), 1.3–7.5%

*Data obtained from questionnaires

**Data obtained from electronic health records

***5-year risk estimated using the INFLUENCE-nomogram using patient-, tumour- and treatment data derived from electronic health records. https://www.evidencio.com/models/show/2238

#### Recurrence risk estimations and risk appraisals

Table 2 displays the recurrence risk estimations and risk appraisals of the participants. Most participants estimate their recurrence risk between 0.1% (1 in 1000) and 2% (1 in 50), which is relatively low. Furthermore, most participants appraise their recurrence risk as neither low nor high (46.5%).

**Table 2:** Recurrence risk estimations and risk appraisals (*n* = *258*)

Recurrence risk estimations	*N* (%)
1 in 1000 (0.1%)	87 (33.7%)
1 in 100 (1%)	81 (31.4%)
1 in 50 (2%)	44 (17.1%)
1 in 25 (4%)	13 (5%)
1 in 10 (10%)	26 (10.1%)
1 in 5 (20%)	7 (2.7%)

Recurrence risk appraisals	*N* (%)
Very low	31 (12%)
Low	81 (31.4%)
Not low/not high	120 (46.5%)
High	25 (9.7%)
Very high	1 (0.4%)

#### Fear of cancer recurrence (FCR)

FCR scores ranges between 6 and 24. The mean FCR score was 14 (on a scale of 6–24) with a standard deviation of 3.6. About three quarters (74.4%) of participants scored above the threshold of 12 indicating elevated levels of FCR [20].

### Accuracy of women’s risk estimations

About half of the participants estimate their risk for LRRs accurately (53.5%), 33.7% of participants underestimated their risk and 12.7% overestimated their risk.

### Relationship recurrence risk estimations and risk appraisals

A Spearman’s rank correlation was computed to assess the relationship between recurrence risk estimations and recurrence risk appraisals. Risk estimations and risk appraisals were only moderately positively correlated *r*(256) = 0.577, *p* = 0.000.

### Factors influencing LRR risk perceptions

#### Factors influencing recurrence risk estimations and recurrence risk appraisals

Spearman’s rank correlations were computed to assess the relationship between several variables and recurrence risk estimations and recurrence risk appraisals (Table 3). No significant correlations were found for recurrence risk estimations. A weak positive correlation was found between radiotherapy and recurrence risk appraisals, *r*(256) = 0.177, *p* = 0.004. A weak negative correlation was found between cure beliefs and recurrence risk appraisals, *r*(256) = − 0.194, *p* = 0.002.

**Table 3:** Associations between variables, recurrence risk estimations, recurrence risk appraisals, and under-, accurate and overestimation of risk for LRR (*n* = *258*)

	Recurrence risk estimations	Recurrence risk appraisals	Accuracy of risk estimations		
			Underestimation (*n* = 87 vs. rest)	Accurate estimation (*n* = 138 vs. rest)	Overestimation (*n* = 33 vs. rest)
Demographics					
Age	− 0.10	− 0.11	0.03	0.13	− 0.23**
Marital status (married/partnership)	− 0.00	0.14	0.01	− 0.01	0.00
Employment status (employed)	0.07	− 0.04	− 0.08	0.02	0.10
Education level	− 0.01	− 0.05	− 0.01	− 0.04	0.07
Illness perceptions					
Believe breast cancer is cured	− 0.11	− 0.19*	0.16	− 0.15	0.00
Believe personal actions affect recurrence risk	0.08	0.09	− 0.01	− 0.07	0.12
Tumour characteristics					
Differentiation grade (Bloom–Richardson)	− 0.06	0.07	0.09	− 0.02	− 0.10
Tumour stage (pT stadium, pathological)	0.01	0.06	− 0.01	0.02	− 0.02
Nodal stage (pN stadium, pathological)	0.04	0.05	− 0.08	0.10	− 0.03
Multifocality	− 0.03	− 0.02	0.06	− 0.11	0.08
Hormone receptor (ER/PR) (positive)	− 0.02	− 0.07	0.00	0.00	− 0.00
Her2neu receptor (positive)	0.05	− 0.02	− 0.03	0.00	0.03
Treatment characteristics					
Type of surgery (lumpectomy vs. mastectomy)	0.00	0.01	− 0.00	0.04	0.06
Chemotherapy	− 0.03	0.09	0.04	− 0.05	0.02
Radiotherapy	0.10	0.18*	− 0.07	0.06	0.00
Hormonal therapy	− 0.08	− 0.00	0.05	0.03	− 0.12
Trastuzumab	0.05	− 0.02	− 0.03	0.00	0.03

*Significant Spearman’s Rank-Order correlation at the *p* < 0.01 level

**Significant Spearman’s Rank-Order correlation at the *p* < 0.001 level

#### Factors influencing over- and underestimation

Spearman’s rank correlations were computed to assess the relationship between several variables and under-, accurate-, and overestimation of LRR risks (Table 3). A weak negative correlation was found for overestimation with age *r*(256) = − 0.234, *p* = 0.000, suggesting that younger women may overestimate their risk more often.

### Association of objective LRR risk and risk perceptions with FCR

No significant correlation was found between the objective LRR risk and FCR (Table 4). However, both recurrence risk estimations and recurrence risk appraisals were significantly positively correlated with FCR. The strongest correlation was found between recurrence risk appraisal and FCR, *r*(256) = 0.389, *p* = 0.000.

**Table 4:** Associations objective LRR risk, risk estimations, and risk appraisals with FCR

	Fear of cancer recurrence
Objective LRR risk	− 0.035
Recurrence risk estimations	0.293**
Recurrence risk appraisals	0.389**

**Significant Spearman’s Rank-Order correlation at the *p* < 0.001 level

A multiple regression analysis was performed to predict FCR from the objective LRR risk, recurrence risk estimations and recurrence risk appraisals. In the regression model, only risk appraisal contributed significantly to FCR (*p* < 0.05).

## Discussion

Dutch breast cancer survivors after treatment for early-stage invasive breast cancer were fairly accurate at estimating their LRR risks. However, they experienced relatively high levels of FCR. With the available data in our study, predictions on recurrence risk estimations, risk appraisals and accuracy of women’s LRR risk perceptions remained difficult. A few weak relations were found: women who did not receive radiotherapy and women who believed their breast cancer was cured appraised their recurrence risk as lower. On the other hand, younger women were more likely to overestimate their risks for LRRs. Recurrence risk estimations and recurrence risk appraisals were significantly associated with higher FCR. However, results of a multiple regression analysis showed that only risk appraisal contributed significantly to FCR.

About half of the women in this study classified their LRR risk accurately (53.5%), 33.7% of participants underestimated their risk, and 12.7% overestimated their risk. This finding is contrary to that of a study by Liu et al. in which only about 17% of all participants estimated their risk accurately. These differences in results may be due to several reasons: the study by Liu et al. included DCIS patients; Adjuvant! Online (a different prediction model used for decision-making about adjuvant treatments) was used to calculate individual risks; participants were asked to estimate their risk on a 0 to 100% scale instead of absolute risk categories (e.g., 1 in 10 or 1 in 100); and participants were asked to estimate their 10-year risk as opposed to the 5-year risk like in our study [6]. However, since 46% still misjudged the LRR risk, it remains important to inform patients about their risks for LRR. This can be supported by the use of prediction models, such as the INFLUENCE-nomogram [4]. Women indicate to welcome this type of information, to support the process of SDM about post-treatment surveillance [5].

In this study, women who did not receive radiotherapy and women who believed their breast cancer was cured appraised their recurrence risk as lower. It is important to note that most respondents in the sample population (81.8%) received radiotherapy. The outcome on radiotherapy is contrary to findings by Liu et al. who found that women who received radiotherapy estimated their risk as lower [16]. A potential explanation for this result could be that patients who did not receive radiotherapy thought that their disease was less severe than that of other women, because they did not ‘need’ radiotherapy as additional treatment. The finding on cure beliefs is consistent with existing literature [13, 14]. Furthermore, younger women were more likely to overestimate their risks for LRRs in our study. This finding is in line with findings by Liu et al. and could be explained by the fact that younger women are less likely to develop breast cancer in the first place and that when they do develop breast cancer they may believe themselves to be at a higher risk of developing a recurrence [15]. Overall, an interesting finding was that risk estimations and risk appraisals remained hard to predict with the variables included in our study. Additional qualitative research on how risk perceptions are established within breast cancer survivors could reveal common misconceptions or gaps in information provision and give directions on how to influence risk perceptions in the future.

Even though our study population had relatively favourable tumour- and treatment characteristics in comparison with other early-stage (M0) breast cancer populations and an on average low risk for LRRs, about 75% of participants scored above the threshold of 12 on the FCR measurement indicating high levels of FCR. This result is in line with another study by Simard et al. and shows the importance of structurally addressing FCR during and after treatment for breast cancer [22].

Recurrence risk estimations and recurrence risk appraisals were only moderately positively correlated with each other. This suggests that these are two different concepts which should both be addressed in patient-provider communication. However, both risk estimations and risk appraisals were significantly associated with FCR. Recurrence risk appraisal turned out to be most predictive for FCR, suggesting that risk appraisal may be more important than the objective (e.g., estimated using a prediction model) or perceived risk when it comes to FCR. Therefore, it is important that in risk communication, attention is given also on how risk is appraised (high or low) by the patient in addition to understanding of the absolute risk. Since the correlations between risk estimations and risk appraisals on the one hand and FCR on the other were only moderate, other factors may also play a role in FCR [13, 15, 17]. Explicit exploration of and communication about a woman’s FCR by clinician is thus recommended. Only then, patients with high levels of FCR can be advised on managing worry or if necessary be referred to suitable (existing) interventions or supportive care [23, 24].

### Limitations and strengths

This study has limitations. The sample population had quite favourable tumour- and treatment characteristics which may make the results less generalisable to populations with patients with higher LRR risks. On the other hand, these favourable characteristics make the results interesting, because of the relatively high levels of reported FCR. Because part of the data was collected during the COVID-19 pandemic this may have influenced risk perceptions and levels of experienced FCR among participants. Another limitation may have been the use of the two domains of the IPQ-BCS questionnaire. Even though a forwards-backwards translation procedure was applied for translation from English to Dutch and internal consistency was good, the Dutch version of the questionnaire was not yet validated and it may have been hard for patients to understand the concepts measured. Strengths of this study are its’ prospective nature; that it included participants with and without clinical levels of FCR and that it was conducted in eight teaching hospitals with dedicated breast centres, covering 15% of the Dutch population and geographically spread over different regions in the Netherlands.

## Conclusion

Although recurrence risk estimations and risk appraisals are significantly associated with FCR, the latter may be more important to address in patient-provider communication in addition to the understanding of absolute risks. Besides risk perceptions, it is important to take other factors into account that influence FCR. Prediction models can support the optimization of information provision and communication about recurrence risks and might help to reduce FCR.

## Data Availability

The data that support the findings of this study are available from the corresponding author upon reasonable request.

## References

[1] Integraal Kankercentrum Nederland (IKNL) (n.d.). NKR cijfers [NCR numbers]. Retrieved from. https://iknl.nl/nkr-cijfers?fs%7Cepidemiologie_id=526&fs%7Ctumor_id=292&fs%7Cregio_id=550&fs%7Cperiode_id=564%2C565%2C566%2C567%2C568%2C569%2C570%2C571%2C572%2C573%2C574%2C575%2C576%2C577%2C578%2C579%2C580%2C581%2C582%2C583%2C584%2C585%2C586%2C587%2C588%2C589%2C590%2C591%2C592%2C593%2C563%2C562%2C561&fs%7Cgeslacht_id=644&fs%7Cleeftijdsgroep_id=677&fs%7Cjaren_na_diagnose_id=687&fs%7Ceenheid_id=703&cs%7Ctype=line&cs%7CxAxis=periode_id&cs%7Cseries=epidemiologie_id&ts%7CrowDimensions=periode_id&ts%7CcolumnDimensions=&lang%7Clanguage=nl. Accessed 26 June 2022

[2] NABON (2012) Breast cancer—Dutch Guideline, version 2.0. Oncoline. Retrieved from. https://www.oncoline.nl/uploaded/docs/mammacarcinoom/Dutch%20Breast%20Cancer%20Guideline%202012.pdf. Accessed 26 June 2022

[3] Witteveen A, Vliegen IM, Sonke GS, Klaase JM, IJzerman MJ, Siesling S (2015). Personalisation of breast cancer follow-up: a time-dependent prognostic nomogram for the estimation of annual risk of locoregional recurrence in early breast cancer patients. Breast Cancer Res Treat.

[4] Völkel V, Hueting TA, Draeger T, van Maaren MC, de Munck L, Strobbe LJA, Sonke GS, Schmidt MK, van Hezewijk M, Groothuis-Oudshoorn CGM, Siesling S (2021). Improved risk estimation of locoregional recurrence, secondary contralateral tumors and distant metastases in early breast cancer: the INFLUENCE 2.0 model. Breast Cancer Res Treat.

[5] Ankersmid JW, Drossaert CHC, van Riet YEA, Strobbe LJA, Siesling S, the Santeon VBHC Breast Cancer Group (2022). Needs and preferences of breast cancer survivors regarding outcome-based shared decision-making about personalised post-treatment surveillance. J Cancer Surviv.

[6] Liu Y, Pérez M, Aft RL, Massman K, Robinson E, Myles S, Schootman M, Gillanders WE, Jeffe DB (2010). Accuracy of perceived risk of recurrence among patients with early-stage breast cancer. Cancer Epidemiol Biomarkers Prev.

[7] Rapport F, Khanom A, Doel MA, Hutchings HA, Bierbaum M, Hogden A, Shih P, Braithwaite J, Clement C (2018). Women’s perceptions of journeying toward an unknown future with breast cancer: the “Lives at Risk Study”. Qual Health Res.

[8] Brandzel S, Rosenberg DE, Johnson D, Bush M, Kerlikowske K, Onega T, Henderson L, Nekhlyudov L, DeMartini W, Wernli KJ (2017). Women’s experiences and preferences regarding breast imaging after completing breast cancer treatment. Patient Prefer Adher.

[9] Hawley ST, Janz NK, Griffith KA, Jagsi R, Friese CR, Kurian AW, Hamilton AS, Ward KC, Morrow M, Wallner LP, Katz SJ (2017). Recurrence risk perception and quality of life following treatment of breast cancer. Breast Cancer Res Treat.

[10] Rothrock NE, Matthews AK, Sellergren SA, Fleming G, List M (2004). State anxiety and cancer-specific anxiety in survivors of breast cancer. J Psychosoc Oncol.

[11] Janz NK, Li Y, Zikmund-Fisher BJ, Jagsi R, Kurian AW, An LC, McLeod MC, Lee KL, Katz SJ, Hawley ST (2017). The impact of doctor–patient communication on patients’ perceptions of their risk of breast cancer recurrence. Breast Cancer Res Treat.

[12] Lee KL, Janz NK, Zikmund-Fisher BJ, Jagsi R, Wallner LP, Kurian AW, Katz SJ, Abrahamse P, Hawley ST (2018). What factors influence women’s perceptions of their systemic recurrence risk after breast cancer treatment?. Med Decis Mak.

[13] Freeman-Gibb LA, Janz NK, Katapodi MC, Zikmund-Fisher BJ, Northouse L (2017). The relationship between illness representations, risk perception and fear of cancer recurrence in breast cancer survivors. Psycho-Oncol.

[14] Kaptein AA, Schoones JW, Fisher MJ, Thong MSY, Kroep JR, van der Hoeven KJM (2015). Illness perceptions in women with breast cancer—a systematic literature review. Curr Breast Cancer Rep.

[15] Liu Y, Pérez M, Schootman M, Aft RL, Gillanders WE, Jeffe DB (2011). Correlates of fear of cancer recurrence in women with ductal carcinoma in situ and early invasive breast cancer. Breast Cancer Res Treat.

[16] Liu Y, Pérez M, Schootman M, Aft RL, Gillanders WE, Ellis MJ, Jeffe DB (2010). A longitudinal study of factors associated with perceived risk of recurrence in women with ductal carcinoma in situ and early-stage invasive breast cancer. Breast Cancer Res Treat.

[17] McGinty HL, Small BJ, Laronga C, Jacobsen PB (2016). Predictors and patterns of fear of cancer recurrence in breast cancer survivors. Health Psychol.

[18] Janz NK, Leinberger RL, Zikmund-Fisher BJ, Hawley ST, Griffith K, Jagsi R (2015). Provider perspectives on presenting risk information and managing worry about recurrence among breast cancer survivors. Psycho-Oncol.

[19] Moon Z, Moss-Morris R, Hunter MS, Hughes LD (2017). Measuring illness representations in breast cancer survivors (BCS) prescribed tamoxifen: modification and validation of the Revised Illness Perceptions Questionnaire (IPQ-BCS). Psychol Health.

[20] Mokkink LB, Prinsen CAC, Patrick D, Alonso J, Bouter LM, de Vet HCW, Terwee CB (2019) COSMIN Study design checklist for patient-reported outcome measurement instruments. https://www.cosmin.nl/wp-content/uploads/COSMIN-study-designing-checklist_final.pdf. Accessed 26 June 2022

[21] Custers JAE, Kwakkenbos L, van de Wal M, Prins JB, Thewes B (2018). Re-validation and screening capacity of the 6-item version of the Cancer Worry Scale. Psycho-Oncol.

[22] Simard S, Thewes B, Humphris G, Dixon M, Hayden C, Mireskandari S, Ozakinci G (2013). Fear of cancer recurrence in adult cancer survivors: a systematic review of quantatitative studies. J Cancer Surviv.

[23] Burm R, Thewes B, Rodwell L, Kievit W, Speckens A, van de Wal M, Prins J (2019). Long-term efficacy and cost-effectiveness of blended cognitive behavior therapy for high fear of recurrence in breast, prostate and colorectal cancer survivors: follow-up of the SWORD randomized controlled trial. BMC Cancer.

[24] Liu J, Butow P, Beith J (2019). Systematic review of interventions by non-mental health specialists for managing fear of cancer recurrence in adult cancer survivors. Support Care Cancer.

